# Immune Responses in Oral Papillomavirus Clearance in the MmuPV1 Mouse Model

**DOI:** 10.3390/pathogens12121452

**Published:** 2023-12-14

**Authors:** Sarah A. Brendle, Jingwei J. Li, Vonn Walter, Todd D. Schell, Michael Kozak, Karla K. Balogh, Song Lu, Neil D. Christensen, Yusheng Zhu, Karam El-Bayoumy, Jiafen Hu

**Affiliations:** 1 The Jake Gittlen Laboratories for Cancer Research, College of Medicine, Pennsylvania State University, Hershey, State College, PA 17033, USA; sblauch1@pennstatehealth.psu.edu (S.A.B.); jenny.jingwei.li@gmail.com (J.J.L.); mkozak1@pennstatehealth.psu.edu (M.K.); kkb2@psu.edu (K.K.B.); ndc1@psu.edu (N.D.C.); 2 Department of Pathology, College of Medicine, Pennsylvania State University, Hershey, PA 17033, USA; lsong@pennstatehealth.psu.edu (S.L.); yzhu@pennstatehealth.psu.edu (Y.Z.); 3 Department of Biochemistry & Molecular Biology, College of Medicine, Pennsylvania State University, Hershey, PA 17033, USA; vwalter1@pennstatehealth.psu.edu (V.W.); kelbayoumy@pennstatehealth.psu.edu (K.E.-B.); 4 Department of Public Health Sciences, College of Medicine, Pennsylvania State University, Hershey, PA 17033, USA; 5 Department of Microbiology and Immunology, College of Medicine, Pennsylvania State University, Hershey, PA 17033, USA; tschell@pennstatehealth.psu.edu

**Keywords:** human papillomavirus, mouse papillomavirus, infections, innate, adaptive, viral clearance, immune responses, oral cavity, sex difference

## Abstract

Human papillomavirus (HPV)-induced oropharyngeal cancer now exceeds HPV-induced cervical cancer, with a noticeable sex bias. Although it is well established that women have a more proficient immune system, it remains unclear whether immune control of oral papillomavirus infections differs between sexes. In the current study, we use genetically modified mice to target CCR2 and Stat1 pathways, with the aim of investigating the role of both innate and adaptive immune responses in clearing oral papillomavirus, using our established papillomavirus (MmuPV1) infection model. Persistent oral MmuPV1 infection was detected in Rag1ko mice with T and B cell deficiencies. Meanwhile, other tested mice were susceptible to MmuPV1 infections but were able to clear the virus. We found sex differences in key myeloid cells, including macrophages, neutrophils, and dendritic cells in the infected tongues of wild type and Stat1ko mice but these differences were not observed in CCR2ko mice. Intriguingly, we also observed a sex difference in anti-MmuPV1 E4 antibody levels, especially for two IgG isotypes: IgG2b and IgG3. However, we found comparable numbers of interferon-gamma-producing CD8 T cells stimulated by E6 and E7 in both sexes. These findings suggest that males and females may use different components of innate and adaptive immune responses to control papillomavirus infections in the MmuPV1 mouse model. The observed sex difference in immune responses, especially in myeloid cells including dendritic cell (DC) subsets, may have potential diagnostic and prognostic values for HPV-associated oropharyngeal cancer.

## 1. Introduction

Alarmingly, the incidence of human papillomavirus (HPV)-induced oropharyngeal cancer (OPC) now exceeds that of HPV-induced cervical cancer [1]. HPV-associated OPC has a high frequency of metastasis at presentation and poor clinical outcomes owing largely to the lack of an effective means of early detection [2,3,4,5]. HPV infection is species-restricted and there is no preclinical model to study HPV in vivo [6]. It was not until 2011 that the first mouse papillomavirus (MmuPV1) was reported [7]. Our laboratory demonstrated that MmuPV1 can infect not only cutaneous tissues but also mucosal tissues, including the oropharyngeal tissues [8]. To provide mechanistic insights on the role of HPV in the development of OPC, our group further established an MmuPV1 oral infection model to mimic HPV infections and associated diseases [9,10,11]. MmuPV1 preferentially infects the base of the tongue, one of the two equivalent sites for HPV infections [9,10]. However, current mouse strains that develop persistent MmuPV1 oral infection and advanced disease are all immunodeficient [8,9,10,11,12,13,14,15], making them unsuitable for studying the complete involvement of the immune system in HPV pathogenesis. 

Immune responses against MmuPV1 infection in cutaneous tissues were previously reported in immunocompetent mice including C57BL/6 (B6) [15,16,17]. MmuPV1 infections in the lower genital tract were also reported in several immunocompetent mouse strains, including B6 together with a panel of gene knockout mice [18,19,20]. Increased viral susceptibility was found in mice with deficiencies in MyD88, CCR6, and Stat1 while B6 mice only showed transient infection [20]. These studies support the hypothesis that prolonged skin and mucosal infections can be established in both innate (e.g., CCR6 and Stat1) and adaptive (e.g., T cells) immune-compromised mice as well as some genetically modified mice [11,15,16,17,18,20,21,22]. Therefore, both innate and adaptive immune responses contribute to the immune control of papillomavirus infections in vivo. However, how and whether different immune pathways play a role in the elimination of virus-infected cells in the oral cavity, particularly in the context of different sexes, remain unknown. The present study utilizes two genetically modified mice (CCR2ko and Stat1ko), aiming to investigate the role of both innate and adaptive immune responses in the clearance of oral papillomavirus using the mouse model.

It is well known that innate immune cells, including myeloid dendritic cells, macrophages, and neutrophils, are first to be recruited to virally infected sites [23,24,25]. These immune cells play a critical role in the link between innate and adaptive immunity and activating adaptive immune responses to counteract local papillomavirus infections [20,26,27,28,29]. Chemokines and their receptors are vital factors for the trafficking of immune cells to these locally impacted tissues [25,30,31,32]. Chemokines expressed by virally infected cells can facilitate the recruitment of these immune cells [32,33]. Two papillomavirus oncoproteins, E6 and E7, play a role in repressing chemokine expression in infected cells and therefore impede the migratory capacity of Langerhans cells during the development of advanced HPV diseases such as cervical cancer [25,31,34]. Dendritic cells (DCs), monocytes, and macrophages are members of the mononuclear phagocyte system that display multiple functions during immune responses. Historically, these cells have been grouped together because although monocytes have their unique functions as mononuclear phagocytic cells, they were also considered as the definitive precursors of macrophages and DCs [35]. CCR2 and CCR6 are key chemokine receptors expressed on circulating DCs as well as local keratinocytes and Langerhans cells that assist DC migration into the tissues at the sites of pathogen invasion [36]. DCs that are rapidly recruited into epithelial tissues play an indirect role by promoting T cell responses that clear viral infections [37]. pDCs have been targeted for treating HPV-associated cancer [38]. Other immune cells, including macrophages and neutrophils, are also important players in innate and adaptive immune responses [39,40,41]. Tumor-infiltrating lymphocytes have been reported valuable in predicting the outcome of HPV-positive OPC [42,43]. Therefore, a deeper understanding of the role of these immune cells in oral infection and control could shed light on developing new diagnosis and treatments for HPV-associated oropharyngeal cancer (OPC). 

Signal Transducer and Activator of Transcription 1 (Stat1) is one of seven mammalian members of the Stat family, a group of transcription factors important in signal transduction [44]. It is best known for its essential role in mediating responses to all types of interferons (IFNs) and is preferentially activated by IFN-γ during viral infections [20,44,45,46,47]. The role of Stat1 in several innate immune pathways has been tested in cervical cancer patients [48] as well as lower genital tract infections in the mouse model [20]. Stat1ko mice were also susceptible to cutaneous MmuPV1 infection and developed visible skin lesions (our unpublished observations). Whether and how these different immune pathways are involved in the control of oral papillomavirus infections remain unclear. Therefore, we included CCR2ko and Stat1ko mice in the current study to compare the immune cell profiles during MmuPV1 clearance in the oral cavity. 

Sex bias has been reported in various infectious diseases [49,50,51], including HPV-associated OPC, although women are just as susceptible to infections as men [52]. It is currently unknown whether a sex difference exists in the immune control of oral papillomavirus infections. The current study investigated oral MmuPV1 infection in both male and female B6 mice and genetically modified mice with deficiencies in both innate and/or adaptive immunity and compared changes in key local immune cell populations during viral clearance of oral papillomavirus using the mouse model.

## 2. Materials and Methods

### 2.1. Viral Infection, Oral Swab, and Oral Tissue Collection

All animal studies were reviewed and approved by the Institutional Animal Care and Use Committee of the Pennsylvania State University College of Medicine and followed NIH guidelines for care and use of animals in research. All mice were housed in the Pennsylvania State University College of Medicine Association for Assessment of Laboratory Animal Care-approved Animal Care Unit. C57BL/6 and a panel of genetically modified mice (CCR2ko, Stat1ko, and Rag1ko) were purchased from the Jackson laboratory. We chose CCR2ko and Stat1ko mice for this study because of their role in innate and adaptive immunity as well as previously published and unpublished studies, suggesting these two genetically modified mice might be more susceptible to MmuPV1 infection than B6 mice [20]. Rag1ko mice were included as the positive control for MmuPV1 infection as these mice with a deficiency in both T and B cell immune responses were as susceptible to MmuPV1 infection as athymic mice [9,15]. The selected mice were generated on the B6 genetic background and so are suitable to compare the changes in viral infection and the immune cell profiling during viral clearance of oral papillomavirus using the mouse model. MmuPV1 infection of mouse oral tissues was achieved using previously described procedures [10]. In brief, 4–6-week-old mice were used for the current study. Female mice received 3 mg Depo-Provera subcutaneously at three days before infection to synchronize them at the diestrus [53,54]. On day 0, all mice were anesthetized with ketamine (100 mg/kg)/xylazine (10 mg/kg), and the base of the tongue epithelium was gently wounded using microneedles [9,10]. All animals were allowed to recover overnight. The following day, each animal was again anesthetized, and virus inoculum (10 µL viral stock solution in saline, 1 × 10^9^ viral DNA in total, an effective dose for infections demonstrated in previous studies) was placed onto the prewounded sites following additional gentle wounding with microneedles. Oral swabs were collected at week 3 from infected mice by holding mice by the scruff, then placing a small plastic brush dipped in saline into the mouth and swirling several times, which was then withdrawn and placed into a collection tube for DNA/RNA extraction and viral DNA/RNA quantification using qPCR [10]. All infected mice were then sacrificed at week four post-viral infection to capture the changes in the infected tongues and corresponding spleens for additional in situ and immune profiling assays.

### 2.2. Viral Load Assessments

Viral titers in the oral swab samples were determined as previously described [10]. The swabs containing oral exfoliated cells were placed into saline in collection vials and total DNA or RNA extracted using standard methods [10]. DNA was amplified using qPCR and primers and probes targeting the E2 region (150 bp amplimer) and quantitated for viral DNA genomes using a standard curve of amplification from purified plasmid DNA containing a single viral genome [10]. The mean and standard errors of mean (SEMs) of the number of viral genomes per sample were plotted for each group.

Viral transcripts in the infected tissues were determined as previously described [54]. Total RNA was extracted from these tissues and reverse transcribed into cDNA for quantitative PCR analysis. The copy numbers were based on 200 ng RNA from each sample (viral RNA E1^E4 transcripts were quantified using primers 5′-TAGCTTTGTCTGCCCGCACT-3′ and 5′-GTCAGTGGTGTCGGTGGGAA-3′ and probe 5′FAM-CGGCCCGAAGACAACACCGCCACG-3′TAMRA). In brief, 200 ng of RNA was reverse transcribed using the RevertAid First Strand cDNA synthesis kit (Thermo-Fisher, Waltham, MA, USA) and 2 µL cDNA was used in the qPCR analysis. Then, 500 nM of each primer and 250 nM probe were used with the Brilliant III qPCR kit (Agilent, Hong Kong, China) with the following qPCR conditions: 95 °C for 3 min, then 40 cycles at 95 °C for 5 s and 60 °C for 10 s on an Agilent AriaMx qPCR machine (Agilent) [54].

### 2.3. Cell Disassociation for Flow Cytometry

The spleen and tongue tissues from infected mice were disassociated by grinding or mincing into small pieces before collagenase disassociation medium was added (Sigma, St. Louis, MO, USA). Briefly, the spleens were ground and pushed through 50 µm mesh. The base of the tongue was minced with a 21 G scalpel blade and suspended in collagenase solution (4% BSA, 4 mg/mL collagenase type I, and 2 mg/mL collagenase type IV in DMEM) and incubated at 37 °C up to 1.5 h with vortexing every thirty minutes. The single-cell suspensions were frozen in FBS with 10% DMSO and stored in liquid nitrogen before flow cytometry analysis.

### 2.4. Flow Cytometry

Antibodies targeting a panel of immune cell markers including CD45 (BV510), CD11b (M1/70, BV421), CD11c (N418, BV785), Ly6C (HK1.4, APC-CY7), Ly6G (1A8, PE-CY7), CD205 (NLDC-145, APC), CD207 (4C7, PE), F4/80 (BM8, BV711) or CD68 (FA-11, BV711), Siglec F (S17007L, PE-CF594), NK1.1 (PK136, Alexa Fluor 488), and Siglec H (551, PerCP/Cyanine5.5) were obtained from Biolegend and used as recommended by the manufacturer. Cell suspensions from either spleens or tongues were prepared and labeled with a panel of multi-color antibodies together with a fixable viability dye (Biolegend, San Diego, CA, USA) to gate the viable cells. Labeled cells were analyzed using the LSR Fortessa in the Flow Cytometry Core of the Penn State College of Medicine. All immune cell populations were gated on live leukocytes (CD45 positive) prior to subset discrimination using FlowJo 10.1r8 software. In brief, viable single leukocytes (CD45^+^) were gated, and myeloid cells (CD11b^+^) and DCs (CD11c^+^) were further analyzed based on different cell surface markers. Two DC population with exclusion of F4-80^+^ cells were further determined based on CD11b expression and were determined as conventional DC2 (cDC2, CD11b^+^) and conventional DC1 (cDC1, CD11b^−^). Dermal DCs (CD11c^+^F4-80^−^CD205^+^) as well as plasmacytoid DCs (CD11b^−^F4-80^−^SiglecH^+^) were further determined. Macrophages (F4-80^+^) and granulocytes (Ly6G^+^) were gated on CD11b^+^ cells with CD11C^+^ excluded. NK cells (NK1.1 positive) from the lymphoid cells were also identified.

### 2.5. Peptide Stimulation and Intracellular Cytokine Assay

MmuPV1 E6/90-99 (KNIVFVTVR) or E7/69-77 (VLRFIIVTG) peptide was synthesized by the Macromolecular Core Facility of the Penn State College of Medicine. Freshly thawed spleen cell suspensions were cultured in complete RPMI medium 10% Hyclone FBS (Cytiva, Marlborough, MA, USA), antibiotics, 50 μM 2-mercaptoethanol (Gibco, Waltham, MA, USA), 1% non-essential amino acids, 1% sodium pyruvate, and HEPES at a final concentration of 2–4 × 10^6^ cells/mL overnight. Cells were left unstimulated or stimulated with 2 mg/mL of MmuPV1 E6/90–99 or E7/69-77 (VLRFIIVTG, Crawley, UK) or no peptide for 4 h together with mouse IL-2 (5 U/mL) and brefeldin A (GolgiPlug, BD Biosciences (Franklin Lakes, NJ, USA), 1/1000 final concentration) at 37 °C with 5% CO_2_. Cells were stained with PE/CY7-tagged anti-mouse CD8α (Clone 53–6.7, Biolegend), permeabilized (fixation/permeabilization buffer, eBioscience, San Diego, CA, USA), intracellular APC conjugated anti-mouse interferon gamma (IFN-γ) (clone XMG1.2, Biolegend) was used, and they were analyzed by flow cytometry on an LSRFortessa. IFN-γ-secreting cells were calculated by gating on a double positive CD8/IFN-γ population [55].

### 2.6. Anti-MmuPV1 Antibody Detection by Enzyme-Linked Immunosorbent Assay (ELISA)

Mouse sera were collected at the termination of the experiment to test for L1 antibodies and antibodies against E4. First, 0.5 µg MmuPV1 L1 virus-like particles (VLPs) in 50 µL PBS were coated on wells of Nunc^®^ MaxiSorpTM microtiter 96-well plates (ThermoFisher Scientific, Waltham, MA, USA) and incubated at room temperature for 30 min. To detect E4, 0.1–0.5 µg of KLH-conjugated MmuPV1 E4 peptide (PKTTPPRRELFPPTPLTQPP, synthesized by China peptide)/well in 50 µL bicarbonate (pH 9.6) buffer was incubated overnight at 37 °C. Coated plates were blocked with PBS/5% dry milk powder for 1 h at room temperature (RT). The ELISA was conducted as reported previously [56]. Each of the serum samples was diluted 1:100 or higher in PBS/5% dry milk and then added to the 96-well plates for 1 h incubation at RT. Plates underwent five washes with washing buffer (0.05% (*v*/*v*) Tween 20 in 1×PBS), before addition of secondary anti-Ig isotypes (IgG1, G2a, G2b, G3, IgM)–alkaline phosphatase (AP) (Southern Biotech, Birmingham, AL, USA) diluted 1:2000 in 5% milk/PBS for 1 h at RT. After five further washes, 100 µL of 1 mg/mL para-nitrophenyl phosphate (pNPP), a substrate for alkaline phosphatase (AP), was added to each well for color development, and absorbance at 405 nm/450 nm was measured using a Fisher Microplate Reader. A mouse monoclonal antibody against MmuPV1 L1 (MPV.A4) was used as the positive control for L1 detection.

### 2.7. Statistical Analyses

Because many analyses involve small sample sizes and data that do not satisfy the necessary normality assumptions, non-parametric methods were applied. In particular, the Mann–Whitney–Wilcoxon rank sum test was applied when comparing values in two groups, and the Kruskal–Wallis test was applied when comparing values in three or more groups. Dunn’s test with a Bonferroni correction was used to implement post hoc testing for the Kruskal–Wallis test. Viral loads among different infected groups and between different sexes were analyzed by Mann–Whitney rank sum test [56]. Statistical significance between different immune cell populations and mouse strains and different sexes was tested using the same statistical program. A *p*-value less than 0.05 was considered significant among different groups. All analyses were performed with R 4.2.2 (R Core Team). To compare the proportions of positive viral samples in infected animals between different groups, we used the Fisher’s exact test to determine whether there is a significant difference between them.

## 3. Results

### 3.1. Viral DNA and RNA Transcripts Were Detected in the Orally Infected Mice

We have shown previously that MmuPV1 can infect both cutaneous and mucosal sites in athymic mice and promote squamous cell carcinoma [8,9,10,11,12,13,14]. Transient MmuPV1 infections of immunocompetent mice as well as genetically modified mice in the skin and lower genital tract were detected by examining infected tissues or longitudinal swabs for viral DNA/RNA and antibodies against viral proteins [15,16,17,18,19,20,21,22]. To determine the susceptibility of B6 mice to MmuPV1 on the tongue and the timeline of viral clearance, we initially tested B6 and Rag1ko mice (both sexes, N = 6). We collected oral swabs at week 2 post-viral infection for viral DNA quantitation. Detectable viral DNA was found in three out of six males, while only one out of six females had a low level of viral DNA. This suggests that male mice might be more susceptible to MmuPV1 infections in the oral cavity (Table 1).

**Figure 1:**
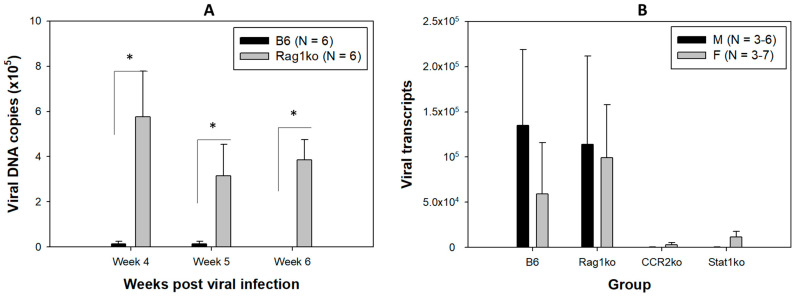
Viral DNA in oral swabs and viral transcripts detected in the infected tongues. We followed viral replication in the infected mice by collecting longitudinal oral swabs and analyzed viral DNA by qPCR. Significantly higher levels of viral DNA were detected in the oral swabs of Rag1ko mice when compared to the infected B6 mice ((**A**), * *p* < 0.05, Mann–Whitney rank sum test) at different time points post-viral infection. No viral DNA was detected in B6 mice at week six post-viral infection. The infected tongues were harvested for viral RNA analysis by qRT-PCR at week four post-viral infection. Viral RNA transcripts were detected in some infected tongues of different mouse strains (Table 2). While more than half of B6 and Rag1ko mice were positive for viral transcripts, two CCR2ko and four Stat1ko mice (out of six each) had very low levels of viral RNA at week four post-viral infection. No significant difference was found between the different groups of animals as well as different sexes ((**B**), *p* > 0.05, the Kruskal–Wallis test).

To determine the timeline of viral clearance, orally infected B6 mice were sacrificed at 4, 5, and 6 weeks post-infection. Viral E1^E4 transcripts, the most abundant viral transcripts produced in productively infected tissues, were used as the indicator for viral clearance in the current study. We detected very low levels of viral DNA in some orally infected B6 mice after week 4 (Figure 1A). We therefore focused on infected mice at week four post-infection in the current study as viral clearance occurs around this time point. We further infected additional B6 and Rag1ko mice (N = 13/group of both sexes) and sacrificed them for viral transcript detection in the infected tongue at week four post-viral infection (Table 2). Similar proportions of infected mice (7 B6 of 13 and 8 out of 13 Rag1ko) exhibited detectable viral RNA in the infected tongue (*p* > 0.05 vs. Rag1ko mice, according to the Fisher’s exact test) at week four post-infection (Table 2).

Together with B6 and Rag1ko mice, two genetically modified mouse strains, chemokine receptor 2 knockout mice (CCR2ko, N = 6/group of both sexes) and Stat1 knockout mice (Stat1ko, N = 6/group of both sexes) were also tested (Table 2). Mice with deficiencies in CCR6 and STAT1 were used to determine the role of the understudied chemokine and interferon pathways in papillomavirus infection in the lower genital tract [20]. Like CCR6, CCR2 is also vital for trafficking of immune cells to the infected tissues [31,32]. Due to the limited availability of CCR6ko mice, we tested CCR2ko mice in the current study because the role of CCR2 in MmuPV1 infection, especially in the oropharyngeal tissues, is unknown [57]. The STAT1 protein helps keep the immune system in balance by controlling the IL-17 pathway [58]. The IL-17 pathway promotes inflammation by mobilizing neutrophils and promotes tissue repair during infection [58]. Stat1ko mice also showed more susceptibility to MmuPV1 infection in both mucosal tissues [20] and cutaneous tissues (our unpublished observation). We hypothesized that deficiencies in either CCR2 or STAT1 proteins inhibit host defense against MmuPV1 infection, leading to increased viral replication and transcription in infected tongues. To our surprise, only two out of six CCR2ko and four out of six Stat1ko mice were positive for viral transcripts in the infected tongues. However, no significant difference in viral positivity among all tested mouse strains was found in the infected tongues (Table 2, *p* > 0.05 vs. Rag1ko mice, according to the Fisher’s exact test).

Rag1ko mice are deficient in both B and T cell immunity and therefore are susceptible to MmuPV1 infection in both skin and mucosal sites like athymic mice [10,22]. Rag1ko mice were used for viral infection control in the current study. To confirm viral infection in tested mice, we monitored viral DNA copies in six B6 and six Rag1ko mice up to week six post-infection by collecting oral swabs. Significantly higher levels of viral DNA were detected in Rag1ko mice when compared to infected B6 mice at all time points (Figure 1A, *p* < 0.05, Mann–Whitney rank sum test). At week six post-infection, no viral DNA was detected in any of the infected B6 mice. In contrast, we found persistent levels of viral DNA in Rag1ko mice over successive time points, a trend similar to what has been observed in athymic mice [9,10,12]. These results confirmed the viral stock used for oral infection was effective for inducing productive infection in immunocompromised mice (Rag1ko).

Viral E1^E4 RNA transcript quantification was conducted for the infected tongues of B6, Rag1ko, Stat1ko, and CCR2ko mice at week four post-viral infection, the active stage of viral clearance in B6 mice. Reduced but not significantly lower viral transcripts were found in infected CCR2ko and Stat1ko mice when compared to those in B6 and Rag1ko mice (Figure 1B, *p* > 0.05, the Kruskal–Wallis test). Therefore, both B6 mice and genetically modified mice with deficiencies in Rag1, CCR2ko, or Stat1ko were susceptible to MmuPV1 infection. However, virally infected cells were cleared in immunocompetent B6 and genetically modified CCR2ko and Stat1ko mice.

### 3.2. Higher Numbers of Dermal Dendritic Cells Were Found in Female B6 Mice

Since both male and female B6 and genetically modified mice showed strong immune control of viral infections leading to viral clearance, we next determined whether both sexes use the same immune strategy to control viral infections. Innate immune responses have been reported to play an important role in controlling HPV infections in humans, especially in the early stage of viral infection [25,41,59]. A previous study using the mouse genital infection model also demonstrated the involvement of several innate immune pathways in MmuPV1 infection [20]. First, we tested local immune cell infiltration by comparing different immune cell subsets using unique CD markers. Dendritic cells (DCs) are major antigen-presenting cells that play a critical role in the link between innate and adaptive immunity [59]. DCs can efficiently prime and activate cellular immune responses and have been targeted for treating HPV-associated cancer [60]. Signaling through CCR2 is vital for the trafficking of immune cells, including DCs, to the infected tissues [36,57], but its role in recruitment of DCs to MmuPV1-infected tongues is unknown. The STAT1 protein helps keep the immune system in balance by controlling the IL-17 pathway which promotes inflammation during infection and promotes tissue repair [58]. However, whether the recruitment of immune cells, including DCs, to fight pathogens locally was inhibited in the absence of STAT1 was also unclear [45,48]. We therefore compared DC recruitment to the infected tongues among CCR2ko, Stat1ko, and B6 mice using a multi-color flow cytometry strategy.

**Figure 2:**
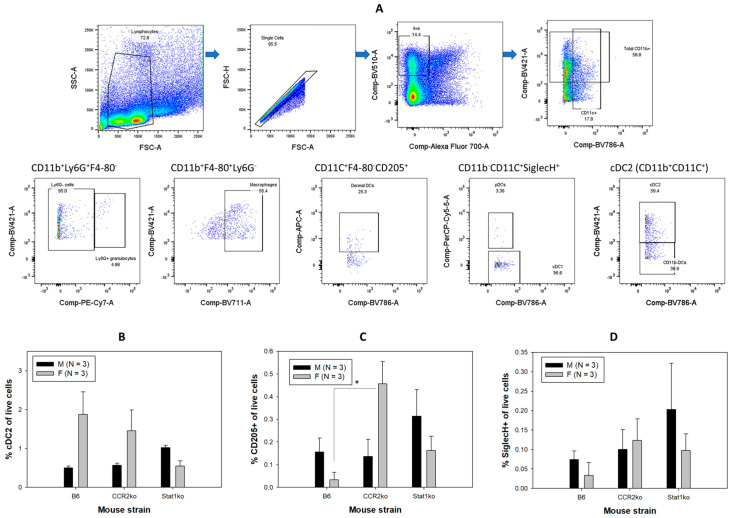
Immune cell profiling using multi-color flow cytometry assay. The schematic flow chart of gating different immune cell populations from flow cytometry assays (**A**). Viable single leukocytes (CD45^+^) from either spleens or tongues were gated for subset discrimination. Total dendritic cells (CD11c^+^F4-80^−^) by exclusion of macrophage (F4-80^+^) cells were further analyzed to determine two dendritic cell populations including cDC1 (CD11b^−^CD11c^+^) and cDC2 (CD11b^+^CD11c^+^). Higher but not significantly higher numbers of cDC2 were found in both B6 and CCR2ko females (based on live cell population) when compared to corresponding males ((**B**), *p* > 0.05, Mann–Whitney rank sum test). Dermal DCs (CD205^+^) of total DCs and plasmacytoid DCs (CD11b^−^SiglecH^+^) were further determined. Macrophages (F4-80^+^) and granulocytes (Ly6G^+^) were identified within the CD11b^+^ cell population. Significantly higher levels of dermal DCs (F4-80^−^CD205^+^) were also found in female CCR2ko mice when compared to corresponding B6 and Stat1ko female mice as well as CCR2ko males ((**C**), * *p* < 0.05, Mann–Whitney rank sum test). No significant difference in pDCs (CD11b^−^SiglecH^+^) was found among all groups even though higher but not significantly higher numbers of pDCs were found in infected Stat1ko males ((**D**), *p* > 0.05, Mann–Whitney rank sum test).

For multi-color flow cytometry, single viable CD45^+^ cells were analyzed based on different CD markers to define immune cell populations. Dendritic cells (DCs) are key antigen-presenting cells for active anti-HPV immune responses [60]. To determine whether there is a sex difference in subtypes of these DC subpopulations during viral clearance, we compared (Figure 2A, viable cells gated on CD11c-positive cells) these two DC populations among the three mouse strains as well as between sexes. We initially analyzed two dendritic cell populations including conventional DC1(CD11b-CD11C^+^, cDC1) and conventional DC2 (CD11b^+^CD11c+, cDC2) [61] in infected tongues of these mice. We further analyzed dermal DCs (CD205+) and plasmacytoid DCs (pDCs, SiglecH^+^) in the CD11b^−^F4-80^+^ cell excluded. These DCs are important antigen-presenting cells to activate T cell immune responses in the control of HPV infections [60]. Higher numbers of cDC2 were found in the tongues of females of B6 and CCR2ko when compared with the corresponding males. However, these differences were not statistically significant (Figure 2B, *p* > 0.05, Mann–Whitney rank sum test).

Significantly higher levels of dermal DCs (CD11b^+^F4-80^−^CD205^+^) were found in tongues of CCR2ko females when compared to the corresponding B6 females (Figure 2C, * *p* < 0.05, Mann–Whitney rank sum test). In contrast, higher but not significantly higher numbers of dermal DCs (CD11b^+^F4-80^−^CD205^+^) were found in the infected tongues of B6 and Stat1ko males when compared to corresponding females (Figure 2C, *p* > 0.050, respectively, Mann–Whitney rank sum test). Similarly, higher but not significantly higher levels of pDCs (CD11b^−^F4-80^−^SiglecH^+^) were found in Stat1ko males when compared to B6 and CCR2ko males (Figure 2D, *p* > 0.05, Mann–Whitney rank sum test). Interestingly, no sex difference in pDCs (CD11b^−^F4-80^−^SiglecH^+^) were recruited to the infected tongues of CCR2ko mice.

### 3.3. Significantly Higher Numbers of Macrophages and Granulocytes Were Recruited to the Infected Tongues of Males

In addition to DCs, other members of the mononuclear phagocyte system including macrophages and neutrophils are also important players in innate immune responses that are recruited to the infected sites [62,63,64]. To determine whether increased frequencies of macrophages and Ly6G^+^ granulocytes were recruited to the infected tongues of MmuPV1-infected mice, we analyzed the CD11b+ population from myeloid cells excluding dendritic cells (CD11b^+^CD11c^−^) in both spleens and tongues in B6 and genetically modified mice according to the gating strategy described in Figure 2A. Dermal DCs (CD11c^+^F4-80^−^CD205^+^) of total DCs and plasmacytoid DCs (CD11c^+^CD11b^−^SiglecH^+^) were further determined. Macrophages (F4-80^+^) and granulocytes (Ly6G^+^) were identified within the CD11b^+^ population.

**Figure 3:**
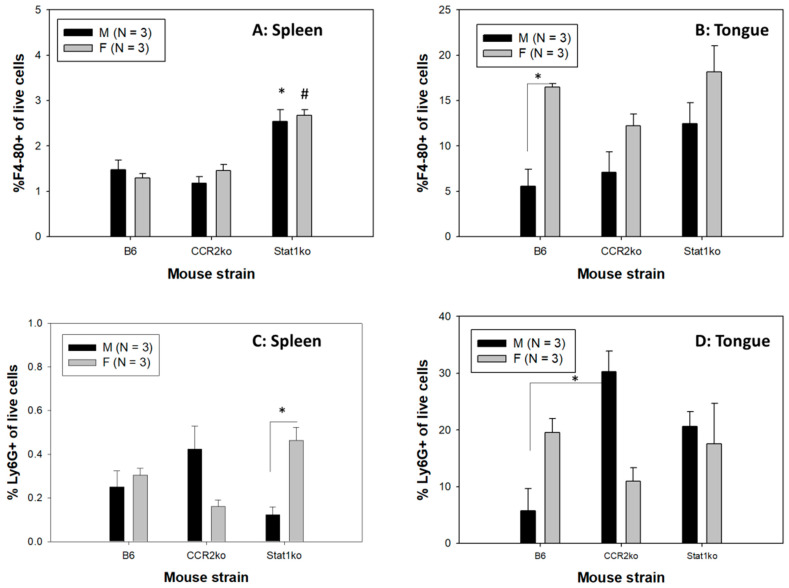
Sex difference in local macrophages and granulocytes was found. Significantly higher levels of macrophages in spleen of both male and female Stat1ko mice (**A**) when compared to corresponding B6 and CCR2ko (* for male, # for female, *p* < 0.05, Mann–Whitney rank sum test). More Ly6G^+^ cells in the spleen were found in Stat1ko females when compared to the corresponding males. Interestingly, higher levels of macrophages were found in infected tongues (**B**) of B6 female mice when compared to corresponding B6 males ((**B**), * *p* < 0.05, Mann–Whitney rank sum test). More Ly6G^+^ granulocytes in the spleen were found in Stat1ko females when compared to the corresponding males ((**C**), * *p* < 0.05, Mann–Whitney rank sum test). A sex difference was not found in the infected tongue of B6 mice for Ly6G^+^ granulocytes ((**D**), * *p* > 0.05, Mann–Whitney rank sum test). Significantly higher levels of Ly6G^+^ granulocytes were found in CCR2ko males when compared to corresponding B6 females (*p* < 0.05, Mann–Whitney rank sum test).

Interestingly, both male and female Stat1ko mice showed higher numbers of macrophages in the spleens when compared to those in B6 and CCR2ko mice (Figure 3A, *p* < 0.05, respectively, Mann–Whitney rank sum test). Significantly higher numbers of F4-80^+^macrophages were detected in infected tongues, but not spleens, of B6 female mice (Figure 3B, *p* < 0.05, respectively, Mann–Whitney rank sum test). Interestingly, both male and female Stat1ko mice showed highest numbers of macrophages in the tongues when compared to those in B6 and CCR2ko mice although they are not significantly different (Figure 3B, *p* > 0.05, respectively, Mann–-Whitney rank sum test). 

Significantly higher levels of Ly6G^+^ granulocytes were found in the spleens of Stat1ko females when compared to those of the corresponding males (Figure 3C, *p* < 0.05, Mann–Whitney rank sum test). CCR2ko males showed the highest levels of Ly6G+ granulocytes in the infected tongues that were significantly higher when compared to B6 males but not when compared to Stat1ko males (Figure 3D, *p* < 0.05 and *p* > 0.05, respectively, Mann–Whitney rank sum test). Lower but not significantly lower levels of Ly6G^+^ granulocytes were found in the infected tongues of CCR2ko females when compared to those of B6 and Stat1ko females (Figure 3D, *p* > 0.05, Mann–Whitney rank sum test). Stat1ko mice, however, failed to show a sex difference in either macrophages or granulocytes (*p* > 0.05, respectively, Mann–Whitney rank sum test).

### 3.4. Similar Numbers of NK Cells Were Found in All Tested Mice

**Figure 4:**
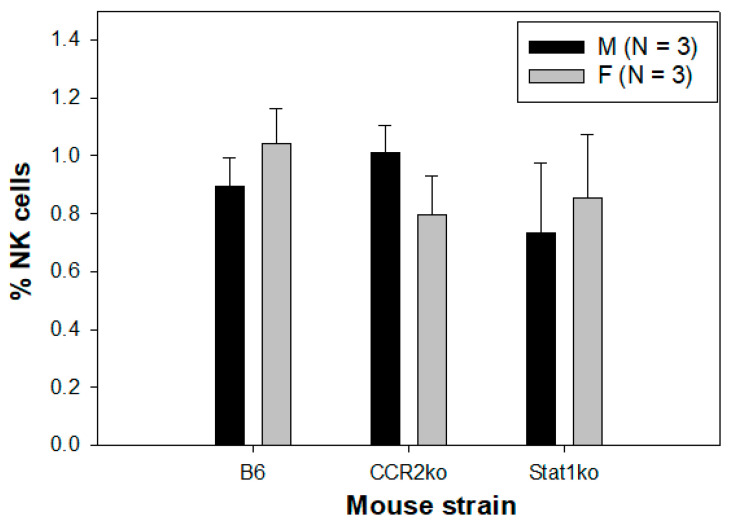
No difference in NK cells was found among different mouse strains. Comparable levels of NK cells were found in infected tongues of B6, CCR2, and Stat1ko mice. Neither sex difference nor strain difference was observed in NK cells in Stat1ko or CCR2ko mice (*p* > 0.05, Mann–Whitney rank sum test).

Natural killer (NK) cells play an important role in innate immune responses to viral infections by producing cytokines upon stimulation and using cytotoxic mechanisms to clear virus-infected cells [64]. NK cells also play a role in HPV infections and pathogenesis [64,65,66]. We therefore tested whether there was any difference in the recruitment of NK cells in infected tongues of these tested mice and whether a sex difference existed as shown in DCs, neutrophils, and macrophages. We failed to detect significant differences in NK cell levels among different mouse strains (Figure 4, *p* > 0.05, Mann–Whitney rank sum test). No sex difference was found in NK cells among B6, Stat1ko, and CCR2ko mice (*p* > 0.05, Mann–Whitney rank sum test), suggesting that accumulation of NK cells in the tongue is not differentially regulated in normal and CCR2- or Stat1-deficient mice during viral control of MmuPV1 infection.

### 3.5. Higher Levels of Anti-MmuPV1 E4 Antibodies Are Detected in Female Mice, Regardless of Strain

**Figure 5:**
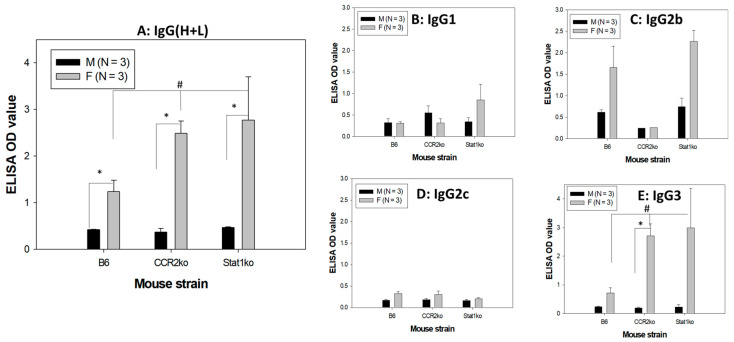
A sex difference in anti-MmuPV1 E4 IgG and isotypes was found. Anti-MmuPV1 E4 IgG antibodies in B6, CCR2ko, and Stat1ko mice post-infection. Significantly higher levels of antibodies against E4 (IgG) were found in females when compared with those of corresponding males of all tested mouse strains ((**A**), * *p* < 0.05, Mann–Whitney rank sum test). Significantly lower levels of E4 IgG were found in B6 females when compared to that of CCR2ko and Stat1ko females ((**A**), # *p* < 0.05, Mann–Whitney rank sum test). Significantly higher levels of antibodies were found in females of CCR2ko for IgG3 (**E**) but not IgG1 (**B**), IgG2b (**C**), and IgG2c (**D**) isotypes. Significantly lower levels of E4 IgG3 were found in B6 females when compared to that of CCR2ko and Stat1ko females ((**E**), # *p* < 0.05, Mann–Whitney rank sum test, * for B6 vs. stat1ko females).

In addition to innate immune responses, adaptive B- and T-cell-mediated immune responses play a key role in viral clearance [23,24,25,26]. Serum conversion in anti-viral protein antibodies in infected animals is additional evidence of productive infection and proper immune responses [18,20]. To test whether infected mice produced B-cell-mediated immune responses against oral MmuPV1 infection, serum samples were harvested at week four post-viral infection for anti-viral protein antibody analyses. We used a peptide of MmuPV1E4, a protein made in abundance, corresponding to the most abundant E1^E4 transcripts in viral RNA detection, after productive MmuPV1 infection. A denatured ELISA was used for the E4 peptide-based ELISA. Interestingly, all infected mice also developed antibodies against MmuPV1 E4 regardless of viral DNA/RNA detection, indicating all the animals were actively infected and responded to MmuPV1 E4 protein. Intriguingly, we observed a sex difference in antibody levels against E4 for all mouse strains. Significantly lower levels of anti-E4 IgG antibodies were found in males when compared with levels in corresponding females (Figure 5A, B6, *p* = 0.0154, CCR2ko, *p* = 0.00643, and Stat1ko, *p* = 0.0724, respectively, Mann–Whitney rank sum test). Significantly higher levels of E4 antibody were also found in both CCR2ko and Stat1ko females when compared to B6 females (Figure 5A, *p* = 0.0281, Mann–Whitney rank sum test) while similarly low levels of E4 antibody were found in males (Figure 5A, *p* = 0.945, Mann–Whitney rank sum test).

To further determine whether there were sex differences in the IgG isotype of these anti-E4 antibodies, we tested five IgG isotypes (IgG1, 2a, 2b, 2c, and 3). Significantly higher levels of E4 IgG3 antibody were found in both CCR2ko and Stat1ko females when compared to B6 females (Figure 5E, *p* = 0.0245 Mann–Whitney rank sum test) while similarly low levels of E4 IgG3 antibody were found in males (Figure 5E, *p* = 0.621, Mann–Whitney rank sum test). Significantly higher levels of IgG3 and other isotype-specific antibodies were found in females when compared with males for CCR2ko mice (Figure 5E, *p* = 0.0243, Mann–Whitney rank sum test). No significant difference was found between males and females for IgG1 (B), IgG2b (C), IgG2c (D), or isotype-specific antibodies (Figure 5B–D, *p* > 0.05, Mann–Whitney rank sum test) even though higher but not significantly higher IgG2b levels were observed in females of B6 and Stat1ko mice. No detectable IgG2a was found in male and female mice.

### 3.6. Anti-MmuPV1 Antibodies Are Skewed toward IgG3 in Females of Additional Inbred Mouse Strains

**Figure 6:**
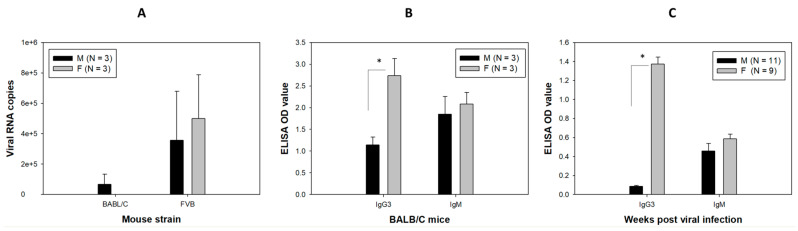
Viral transcripts (**A**) in the infected tongues and anti-MmuPV1 E4 IgG (**B**,**C**) in the sera of MmuPV1-infected BALB/C and FVB mice. BALB/C and FVB mice were tested for infection in the tongue. Similar levels of viral transcripts were found in both male and female mice for both strains (**A**). Similar to what we observed in other mice, significantly higher levels of anti-MmuPV1 E4 isotype IgG3 were found in females of BALB/c ((**B**), * *p* = 0.0395, Mann–Whitney rank sum test) and FVB ((**C**), * *p* < 0.01, Mann–Whitney rank sum test) when compared to the corresponding males. No significant difference was found in anti-MmuPV1 E4 isotype IgM between mouse strains.

To determine whether a similar sex difference in anti-viral E4 antibody occurs in other common immunocompetent mouse strains that have been reported previously for MmuPV1 infection in other tissues [48,49,50,51,67,68,69], we infected BALB/c and FVB strains (N = 6/group, both males and females) at the base of the tongue as we did for other mice described above. The mice were sacrificed at week four post-infection. We detected viral transcripts in one BALB/C and five FVB of six tested mice, respectively (Figure 6A). Interestingly, significantly higher levels of IgG3 E4 antibodies were found in both BALB/C (Figure 6B) and FVB (Figure 6C) mice, suggesting that the anti-MmuPV1 E4 IgG3 isotype was preferentially generated in females of tested mouse strains, irrespective of viral detection in these mice.

### 3.7. Comparable T-Cell-Mediated Immune Responses to MmuPV1 E6 and E7 in Both Sexes of Infected B6 Mice

**Figure 7:**
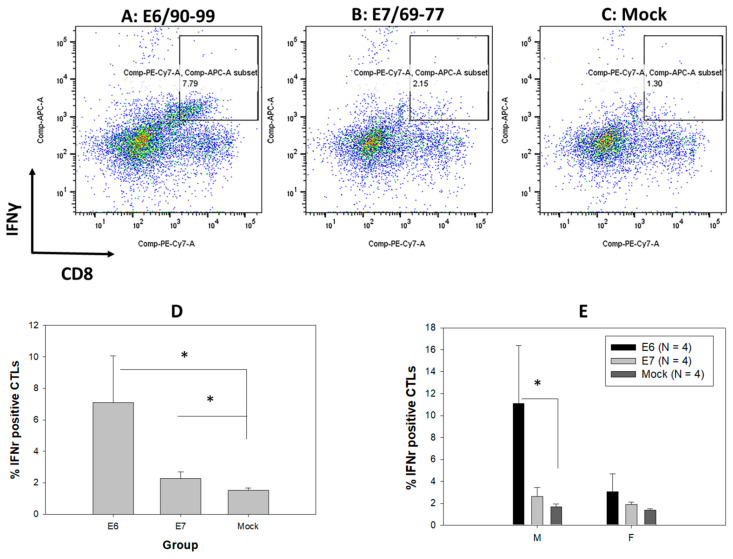
Cytotoxic T cells against anti-MmuPV1 E6 and E7 were detected in infected B6 mice. Anti-MmuPV1 CD8 T cells (PE/CY7-tagged) that release interferon gamma (APC tagged) after MmuPV1 E6/90-99 (KNIVFVTVR) (**A**), E7/69-77 (VLRFIIVTG) (**B**), or mock (**C**) peptide stimulation in splenocytes from both male and female B6 mice. Significantly higher numbers of CD8 T cells to E6/90-99 were detected when compared to E7 and mock-infected B6 mice ((**D**), * *p* < 0.05 vs. mock, the Kruskal–Wallis test). When separating the data based on sex, a significant difference was found in male B6 mice to E6/90-99 ((**E**), * *p* < 0.05 vs. mock, the Kruskal–Wallis test).

A previous study revealed T-cell-mediated immune responses in E6- and E7-vaccinated mice and MmuPV1-infected B6 mice during tumor regression [15]. To further examine whether there is a sex difference in T-cell-mediated immune responses in our oral-MmuPV1-infected mice, we used two reported peptides, MmuPV1 E6/90-99 (KNIVFVTVR) and E7/69-77 (VLRFIIVTG), to stimulate immune cells from spleens and gated on a CD8-positive T cell population that produced intracellular cytokine IFN-γ (Figure 7A–C). In agreement with the previous study, infected wild type B6 mice revealed a CD8 T cell population specific for E6 and E7 peptides in which E6 is the dominant antigen in infected C57/BL6 mice [15,20]. Increased levels of E6-specific CD8 T cells were found in the infected B6 mice after in vitro stimulation (Figure 7D, *p* < 0.05 vs. mock, the Kruskal–Wallis test) when compared to E7-specific and mock. A significant difference was found in E6-specific CD8 T cells vs. mock in male B6 mice (Figure 7E, *p* < 0.05 vs. mock, the Kruskal–Wallis test).

## 4. Discussion

To the best of our knowledge, this is the first study to simultaneously characterize both innate and adaptive immune parameters in a mouse papillomavirus oral infection model during viral clearance. This study compares changes in both innate and adaptive immune responses in immunocompetent mice (B6) and two genetically modified strains with deficiencies in innate (CCR2, Stat1) and/or adaptive immunity (Rag1). We detected a striking sex difference, suggesting males and females used different immune pathways to control and clear oral papillomavirus. These findings provide insights to inform clinical studies such that developing diagnoses based on gender may improve the outcome of HPV-infected patients.

Intriguingly, across species, females tend to develop a stronger innate and adaptive immune response to contagions [51]. Sex difference in immune responses to infectious diseases has been reported [70,71]. HPV infection and associated OPC also showed higher incidence in males when compared with females [1]. We hypothesize that a sex difference in immune response to HPV infection leads to different disease outcomes. MmuPV1 preferentially infects the base of the tongue of immunocompromised athymic nude mice and induces intraepithelial dysplasia and oral squamous cell carcinoma that mimics HPV-associated OPC [9,10]. However, MmuPV1 only caused transient infection in immunocompetent mice, and the infection was cleared within four weeks post-infection [18]. To better understand how healthy hosts clear HPV infection and whether there is a sex difference in immune control underlying viral clearance, we tested wild type C57BL/6 mice and three genetically modified mouse strains that are deficient in either innate or adaptive immune responses (Stat1, CCR2, as well as Rag1ko) [20]. Consistent with our previous findings in athymic nude mice with T cell deficiency [7], not all the animals were positive for viral DNA in the oral swabs or viral transcripts in the infected tongues before week four post-infection, including Rag1ko mice with deficiencies in both T and B cells [7]. Our study further confirmed the critical role that T cells play in papillomavirus clearance [11,15,17]. Interestingly, mice with deficiencies in Stat1 and CCR2 did not show an increase in viral load when compared with the wild type mice at week four post-viral infection in our current study. To confirm that the viral infection was effective for the dose we applied to these mice, we followed additional B6 and Rag1ko mice up to six weeks post-viral infection (Figure 1A). We detected persistent viral DNA levels in the oral swabs of all tested Rag1ko but B6 mice, suggesting the virus was infectious but complete viral clearance might occur in some infected B6 mice. This finding agrees with HPV epidemiological observation as most people clear infection within 1–2 years post-infection [1]. Compared to B6 mice, mice with deficiencies in Stat1 and CCR2 did not show an increase in viral load at this time point. However, we did observe increased susceptibility of Stat1ko mice to tail infection with visible lesions at week 10 post-infection (unpublished observations). Whether a longer incubation of viral infection in CCR2ko and/or Stat1ko mice would increase viral detection is unclear. We postulate that these genetically deficient mice might take advantage of redundant immune mechanisms to clear viral infection, for example, by promoting the recruitment of macrophages and neutrophils, as we determined in this study. Our previous study also found delayed but complete viral clearance in CD28ko mice [22]. Future studies will uncover additional mechanisms underlying the immune control of papillomavirus using these unique genetically modified animals.

The sex difference in myeloid immune cells in infected tongues in our tested mice is striking. Innate immune cells, including myeloid cells (monocytes, macrophages, neutrophils, and dendritic cells) and lymphoid cells (natural killer cells), are recruited to virally infected sites and are critical for disease control [60]. Among dendritic cell subtypes, slightly higher numbers of cDC2 were found in both B6 and CCR2ko females when compared to corresponding males (Figure 2B). On the other hand, dermal dendritic cells (CD205+, dDCs) and plasmacytoid dendritic cells (SiglecH^+^ pDCs) were two important cells also tested in our study. Slightly higher levels of dDCs were found in males when compared to corresponding females for B6 mice (Figure 2C,D). Stat1 is a key immune stimulator that impacts interferon activation during viral infections. Interestingly, Stat1ko male but not female mice showed slightly higher levels of both dDCs and pDCs when compared to B6 and CCR2ko male mice (Figure 2C,D). Opposite to B6 mice, female CCR2ko mice promoted the recruitment of dermal DCs into infected tongues. These findings suggest that CCR2ko male and female mice might promote the recruitment of different immune cells to the infected tongues to aid the clearance of local viral infections.

Several Fc receptors, including the activator receptor, FcγRIIa, and the inhibitory receptor, FcγRIIb, are expressed on plasmacytoid dendritic cells (pDCs) in about 10% of healthy individuals [56]. FcγRIIa plays a dominant role in facilitating the phagocytosis of immune complexes containing “self” or viral nucleic acids by pDCs, resulting in sensing of those nucleic acids by Toll-like receptors and subsequent pDC activation [56,57]. We detected increased levels of dDCs but not pDCs in the infected tongue of female CCR2ko mice but not in B6 and Stat1ko mice, suggesting that dermal DCs but not pDCs might play a more important role in controlling early infections in CCR2ko female mice. For other immune cells, we observed increased numbers of Ly6G^+^ granulocytes and macrophages in B6 females when compared to corresponding males (Figure 3B,D). In contrast, a sex difference was only found in Ly6G^+^ granulocytes of CCR2ko male tongues when compared to that of B6 males (Figure 3D). A previous study suggested that activation of interferon-α-dependent NK cells and pDCs by IgG1/IgG3 may play a key role in controlling HIV infections [57]. In our study, NK cells did not seem to play a significant role in viral clearance. Therefore, the role of both CCR2ko pathways and their impact on different DCs, macrophages, and granulocytes in the control of papillomavirus infections require further investigation.

We did not detect a correlation between viral DNA/RNA detection with anti-MmuPV1 immune responses in our tested animals. For example, low levels of viral transcripts were detected in CCR2ko mice, and anti-viral immune responses were evident in these mice. CCR2ko male mice showed an elevated level of granulocytes in the infected tongue tissues when compared to corresponding females (Figure 3D) but no sex difference in anti-MmuPV1E4 IgG2b antibodies in this mouse strain as shown in B6 and Stat1ko mice (Figure 5C). Chemokine receptors, including CCR2 and CCR6, are expressed on various cell types, including CD4 and CD8 T cells [36]. A recent study demonstrated that cancer cell CCR2 orchestrates suppression of the adaptive immune response [57]. CCR6ko mice also showed more severe pathology after MmuPV1 infection in the vaginal tract [20]. Due to the limited availability of CCR6ko mice, we tested CCR2ko mice in the current study. Additional studies are warranted to further determine how these different myeloid cell populations, without CCR2 and CCR6, impact HPV infections and pathogenesis.

Our observation that the IgG antibody level against MmuPV1 E4 is significantly higher in female mice when compared with males in all tested mouse strains is intriguing (Figure 5). In the original test, we synchronized the estrus stage of females using Depo as we used in previous studies [72]. Previous studies demonstrated that local IgG levels were highest in mice post-infection or -immunization at the diestrus stage [73,74]. We did not monitor oral IgG antibodies in our study. However, we did compare infected B6 mice with Depo or without Depo treatment and observed a similar pattern in anti-MmuPVE4 IgG, suggesting this difference was not associated with the estrus cycle in our mice. Importantly, we did not observe a sex difference in anti-MmuPV1 L1 antibody levels or anti-MmuPV1E4 IgM isotype in these tested animals. Among the panel of IgG isotypes, antigen-specific IgG2c in B6 mice is the most efficient IgG subclass for anti-pathogen FcR-mediated effector functions and the predominant isotype induced by viral infections [75,76,77,78,79]. We did observe slightly higher IgG2c levels for anti-MmuPV1E4 but they were not as significant as IgG2b and IgG3 in all tested mice, suggesting that IgG3 is the predominant isotype of anti-E4 antibodies in MmuPV1-infected mice. IgG3 is the only IgG that forms non-covalent oligomers with increased functional affinity to polyvalent antigens and efficiently agglutinates erythrocytes [80]. IgG3 also triggers the complement cascade. Passive immunization with IgG3 is highly protective due to the unique properties of this subclass [81]. IgG3 has been reported to be correlated with chronic bacterial sinusitis [82] and viral infections [83,84]. The sex difference was not due to the circulating IgG3 in these tested mice as similar levels of IgG3 were found in both non-infected and infected male and female mice. Whether the observed higher levels of IgG3 anti-MmuPV1 E4 antibodies play a role in improved control of MmuPV1 infections in females remains unclear.

Preferences for Th1 responses in C57BL/6 mice and Th2 responses in BALB/c mice are well established in the literature [81]. Our genetically modified mice in this study are all based on the B6 background and therefore the dominant IgG3 isotype anti-viral antibody response may be the result of IFN-γ release that induces isotype switching to IgG2a/c and IgG3 [85]. Interestingly, a same sex difference of higher anti-E4 IgG3 isotype-specific antibodies was also found in female BALB/c and FVB strains, suggesting a common humoral response against papillomavirus infection in the mouse model. We postulate that male mice may have a deficiency in recognizing, processing, or presenting the viral E4 antigen, leading to the decreased levels of IgG2b and IgG3 antibodies to E4 after viral infections. However, the E4 antibody is not associated with viral clearance in these mice as no significant sex difference in viral transcripts was found at week four post-viral infection. Antibodies against multiple early genes were more sensitive and specific for screening of HPV-associated OPC [86]. Monitoring serum levels of immunoglobulins against both early and late proteins has been reported in both natural HPV-infected populations and vaccinated people [86,87,88]. Our study suggests monitoring antibodies to different viral proteins can add value to early diagnosis.

Comparable anti-E6 and -E7 CD8 T cell responses in male and female B6 mice were observed in our study. Our finding further supports the key role of T-cell-mediated immune responses in HPV control and clearance in the oral cavity [2]. Similarly, anti-MmuPV1 E6 and E7 T cells were found to be the key players for MmuPV1 clearance in cutaneous and genital tissues [13,14,15,16,18,20,21,89]. MmuPV1 E6 and E7 oncogenes share similar functions to those expressed by HPVs [11,90]. Like HPV16E6 as the dominant antigen involved in adaptive immunity in humans [91], MmuPV1 E6 has been identified as the immune-dominant antigen and relevant correlate of anti-viral immunity in mice clearing MmuPV1 infections in other tissues as well as the infected tongues. Therefore, our findings agree with those reported in human studies and further support the key role of T-cell-mediated immune responses in viral clearance in both male and female mice.

**Figure 8:**
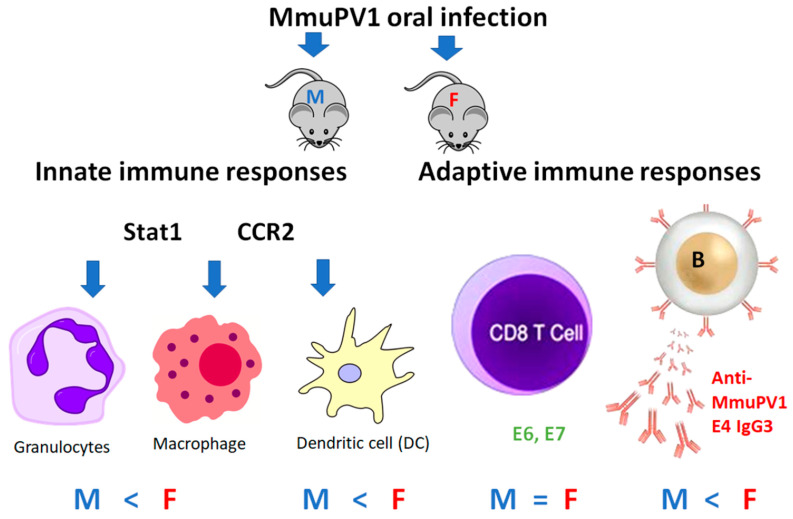
The schematic model for immune responses during viral clearance in the oral MmuPV1 infection mouse model. Several key innate immune cells that are involved in viral clearance of males (M) and females (F) are shown. Both Stat1 and CCR2ko mice showed increased dermal dendritic cell population in the infected tongues when compared with B6 mice. In contrast, CCR2ko male mice recruited increased numbers of ly6G+ granulocytes to the infected tongues than corresponding B6 and Stat1ko males. A similar pattern of sex difference in macrophages and neutrophils was found in B6 and Stat1ko mice. Both sexes elicited similar levels of CD8 T-cell-mediated immune responses after MmuPV1 infection. Intriguingly, significantly higher levels of anti-E4 IgG (especially IgG3 subtype) were found in females irrespective of mouse strain with increased levels in both CCR2 and Stat1ko female mice. Symbols: “>” higher; “<” lower; and “=” comparable levels of immune cell population/responses were detected between males (M) and females (F).

In summary, we report here for the first time an interesting sex difference in innate and adaptive immune responses to papillomavirus infection and clearance in the MmuPV1 model. As depicted in Figure 8, we report the local immune responses of MmuPV1-infected oropharyngeal tissues of B6 mice and genetically modified mice with deficiencies in innate and/or adaptive immunity during viral clearance. The Rag1ko mice, which have T and B cell deficiencies, failed to clear the viral infection (Figure 1A). However, other tested mouse strains with either innate or adaptive immune deficiencies were able to clear the infection by modulating the local myeloid cell population, including dendritic cells, macrophages, and neutrophils, as well as anti-E4 IgG3 antibodies during viral clearance [20,22].

Many co-factors, including UV irradiation [92] and the tobacco carcinogen dibenzo[def,p]chrysene [12], have been reported to promote cancer development in the mouse papillomavirus model. Interestingly, these co-factors also showed immune suppression in treated animals. The immune cell populations influenced by these co-factors are unknown. To further define HPV-associated sex bias in OPC occurring in males, future studies will focus on testing the impact of estrogen receptors on differential immune cell recruitment. The impact of sex differences in myeloid cells and antibody responses to MmuPV1 E4 protein warrants additional investigation. There are a few limitations to consider in the interpretation of our results. First, viral clearance in different infected animals seemed to happen at different time points before week 4, therefore the changes observed at week 4 for all animals were not completely synchronized. The infected tongues that had undetectable viral transcripts in some of the tested mice may indicate that the virus was cleared before week four post-infection or viral transcripts were undetectable due to low levels of infection in these animals. Second, we used a small sample size for some mouse strains. Due to the individual differences, the findings in some experiments may be biased and impact our interpretation. This notwithstanding, our conclusions are plausible, especially the sex differences in the immune responses to viral clearance.

In conclusion, our work demonstrates for the first time that the sex bias in HPV-induced OPC may be related to the sex difference in both innate and adaptive immune responses in viral control. For future directions, defining the subsets of local immune cells, particularly the myeloid cells such as DCs and subsets observed in the infected tissues of different sexes, is needed. These studies will unravel the complex inter-relationships in viral–host interaction and provide additional insights to inform clinical studies for the improved diagnoses of HPV-infected patients based on gender. These proposed future studies will be crucial to lay a solid foundation for further modeling of immune responses and developing effective strategies for interception and prevention of HPV-associated OPC patients.

## Figures and Tables

**Table 1:** Viral DNA detection in the oral swabs of B6 mice at week two post-infection.

Strain	Sex	Viral Positive Mice /Infected Mice	Viral DNA Copies (×10^5^)
B6	M	3/6	24, 7.2, 7.2, 0, 0, 0
	F	1/6	0.84, 0, 0, 0, 0, 0

**Table 2:** Summary of infected animals and viral transcript positivity in the infected tongues.

Strain	Sex	Viral RNA Transcripts (×10^5^)	Positive/Infected Mice (Males + Females)
B6	M	4, 4, 0, 0, 0, 0.1	7/13
	F	4, 0.04, 0, 0, 0.105, 0, 0	
Rag1ko	M	0, 0, 0.6, 6, 0.038, 0.2	8/13
	F	0.105, 0.1075, 0, 4, 0.5, 2.25, 0	
CCR2ko	M	0, 0, 0.009	2/6
	F	0, 0, 0.08	
Stat1ko	M	0, 0.003, 0.003	4/6
	F	0.15, 0, 0.2	

## Data Availability

The data supporting the finding of this study are available from the corresponding author upon request.
